# Lipoxin A_4_ Receptor Stimulation Attenuates Neuroinflammation in a Mouse Model of Intracerebral Hemorrhage

**DOI:** 10.3390/brainsci12020162

**Published:** 2022-01-26

**Authors:** Risa Futokoro, Masanori Hijioka, Moe Arata, Yoshihisa Kitamura

**Affiliations:** 1 Laboratory of Pharmacology and Neurobiology, Collage of Pharmaceutical Sciences, Ritsumeikan University, 1-1-1 Noji-Higashi, Kusatsu 525-8577, Japan; futokoro@mukogawa-u.ac.jp (R.F.); 0404.moe.0404@gmail.com (M.A.); yo-kita@fc.ritsumei.ac.jp (Y.K.); 2 Department of Pharmacology II, School of Pharmacy and Pharmaceutical Sciences, Mukogawa Women’s University, 11-68 Koshien Kyuban-cho, Nishinomiya 663-8179, Japan; 3 Department of Neurocognitive Science, Institute of Brain Science, Nagoya City University Graduate School of Medical Sciences, 1 Kawasumi, Mizuho-cho, Mizuho-ku, Nagoya 467-8601, Japan

**Keywords:** intracerebral hemorrhage, neuroinflammation, lipoxin A_4_, neutrophil, microglia

## Abstract

Intracerebral hemorrhage (ICH) is caused by the rupture of blood vessels in the brain. The excessive activation of glial cells and the infiltration of numerous inflammatory cells are observed during bleeding. Thrombin is a key molecule that triggers neuroinflammation in the ICH brain. In this study, we focused on lipoxin A_4_ (LXA_4_), an arachidonic acid metabolite that has been reported to suppress inflammation and cell migration. LXA_4_ and BML-111, an agonist of the LXA_4_ receptor/formyl peptide receptor 2 (ALX/FPR2), suppressed microglial activation; LXA_4_ strongly inhibited the migration of neutrophil-like cells in vitro. ALX/FPR2 was expressed on neutrophils in the ICH mouse brain and the daily administration of BML-111 attenuated the motor coordination dysfunction and suppressed the production of proinflammatory cytokines in the ICH mouse brain. On the other hand, BML-111 did not show a significant reduction in the number of microglia and neutrophils. These results suggest that systemic administration of ALX/FPR2 agonists may suppress the neuroinflammatory response of microglia and neutrophils without a change in cell numbers. Additionally, their combination with molecules that reduce cell numbers, such as modulators of leukotriene B_4_ signaling, may be required in future studies.

## 1. Introduction

Intracerebral hemorrhage (ICH) is mainly caused by hypertension and consists in the rupturing of blood vessels and leakage of blood into the brain parenchyma [1]. Severe prognoses such as motor dysfunction, sensory paralysis, and impaired consciousness are observed in patients with ICH. These lower their quality of life, with no current medical treatments being highly effective [2,3]. Much research has reported the inhibition of hematoma expansion, promotion of hematoma resolution, suppression of inflammatory reactions, and inhibition of neurodegeneration in an ICH rodent model; however, its translation to ICH patients has not been achieved [4]. Therefore, the development of novel therapeutic targets is required. In this research, we focused on neuroinflammation after ICH onset. An increasing amount of evidence has shown that microglia and macrophages play a pivotal role in pathological events in the ICH rodent model [5,6]. Microglia/macrophages release injurious molecules such as proinflammatory cytokines, reactive oxygen species (ROS), and matrix metalloproteinases (MMPs) in the ICH brain [7,8]. Many molecules that induce the activation of microglia/macrophages have also been identified. Ferrous ions (Fe^2+^) and heme are released by hemolysis and contribute to microglial activation [9]. Thrombin, a blood coagulation factor, and high mobility group box-1 protein, derived from damaged cells, as well as ATP, also induce excessive inflammatory phenotypes in microglia/macrophages [10,11,12]. On the other hand, recent studies have shown the importance of circulating blood cells [13]. In particular, neutrophils, the most abundant granulocyte type, promote the exacerbation of tissue damage after ICH. Neutrophil depletion by the injection of anti-polymorphonuclear neutrophil (PMN) antibodies attenuated axonal damages [14]. Our recent research focused on axonal injury after ICH, because damage to the internal capsule, composed of axonal fibers of the cortico-spinal tract (CST) worsened the prognosis in the ICH mouse model [15,16]. Interestingly, the neutrophil–lymphocyte ratio, evaluating systemic inflammation, was correlated with prognosis in the patient with ICH [17]. This background suggested that the suppression of not only microglia/macrophages, but also of neutrophils, will be a promising therapeutic strategy for ICH through the suppression of inflammation and axonal injury.

Chemoattractants play an important role in cell–cell contacts under physiological and pathological conditions [18]; many chemokines are released and involved in ICH pathogenesis. The C-C motif chemokine 2 (CCL2)/monocyte chemoattractant protein 1 (MCP-1)-C-C chemokine receptor type 2 (CCR2) axis is reported as the major factor promoting monocyte recruitment to the hemorrhaged brain [19]. We revealed that C-X-C motif chemokine ligand 2 (CXCL2) was increased in the ICH brain and involved in motor dysfunctions after ICH [20]. Leukotriene B_4_ (LTB_4_), an arachidonic acid (AA) metabolite, is a chemoattractant known to strongly promote lymphocyte migration activity [21]. Comprehensive gene expression analyses in perihematomal tissue of ICH patients revealed the increased expression of ALOX5 encoding 5-lipoxygenase, a rate-limiting enzyme in LTB_4_ production [22]. We and another group investigated the contribution of LTB_4_ in the ICH brain. LTB_4_ expression was increased in the ICH mouse brain, with LTB_4_ being involved in tissue damage [23,24]. Furthermore, microglia partially carried on the production of LTB_4_, and microglia-released LTB_4_ induced microglial activation and the infiltration of neutrophils [25]. These data suggested that lipid metabolism in the early phase of ICH is important to regulate microglia, macrophage, and neutrophil responses.

Lipid metabolism has physiological significance in nutrient intake, cell membrane structure maintenance, and endocrine system regulation. Metabolic changes in lipid mediators were reported in central nervous system (CNS) diseases such as Alzheimer’s disease, Parkinson’s disease, and stroke [26,27,28]. AA is a major lipid mediator and precursor of prostaglandins (PGs) and leukotrienes (LTs) [29], which are mainly involved in inflammatory reactions in stroke [30,31]. Recent research has shown that other AA metabolites have anti-inflammatory properties. Lipoxin A_4_ (LXA_4_) is produced from AA in reactions catalyzed by 5-lipoxygenase and 12/15-lipoxygenase [30,31]. Some reports have shown that LTB_4_ and LXA_4_ are produced by a similar pathway but have opposite effects, being inflammatory and anti-inflammatory lipid mediators, respectively [32]. LXA_4_ binds the lipoxin receptor (ALX)/formyl peptide receptor 2 (FPR2) and mediates the resolution of inflammation in bowel and lung disease and ischemic stroke; however, there is little knowledge of its role in hemorrhagic stroke [33,34,35]. In this research, we focused on the function of LXA_4_-ALX/FPR2 signaling in the ICH mouse brain and investigated its efficacy as a therapeutic target for ICH.

## 2. Materials and Methods

### 2.1. Cell Culture

Murine BV-2 microglial cells were purchased from Banca Biologica e Cell Factory (Genova, Italy) and maintained in Dulbecco’s modified Eagle’s medium (DMEM) (FUJIFILM Wako Pure Chemical Corp., Osaka, Japan) containing 10% fetal bovine serum (FBS) (Thermo Fisher Scientific Inc., Waltham, MA, USA), 100 U/mL penicillin G, and 100 mg/mL streptomycin (FUJIFILM Wako) under a humidified atmosphere with 5% CO_2_ at 37 °C. For drug treatment, BV-2 cells were seeded on 35 mm cell culture dishes at 3.0 × 10^4^ cells/cm^2^ for 24 h, followed by replacement of culture medium with serum-free DMEM. After 2 h incubation, ALX/FPR2 agonists were applied to cultures in serum-free DMEM. 5S,6R,15S-trihydroxy-7E,9E,11Z,13E-eicosatetraenoic acid (LXA_4_) and 5(S),6^®^-7-trihydroxymethyl Heptanoate (BML-111) were purchased from Cayman Chemical Co. (Ann Arbor, MI, USA). Cells were treated with 30 U/mL thrombin (Sigma-Aldrich, St. Louis, MO, USA) 1 h after treatment with LXA_4_ (100 nM) or BML-111 (500 nM).

The human promyelocytic leukemia cell line HL-60 was provided by the RIKEN BRC through the National Bio-Resource Project of the MEXT, Japan, and maintained in Roswell Park Memorial Institute (RPMI) 1640 medium (FUJIFILM Wako) supplemented with 10% FBS, 100 U/mL penicillin G, and 100 mg/mL streptomycin under a humidified atmosphere with 5% CO_2_ at 37 °C. HL-60 cells were cultivated for 5 days with 1% all-trans retinoic acid (ATRA)-containing medium to differentiate them into neutrophil-like cells.

### 2.2. Animals

All procedures were approved by the ethics committees on animal experiments of Ritsumeikan University and Nagoya City University. Animals were treated in accordance with the Guidelines of the United States Public Health Service’s Policy on Humane Care and Use of Laboratory Animals. Male C57BL/6J mice at 8 weeks of age weighing 21 to 28 g were purchased from Japan SLC Inc. (Shizuoka, Japan). Animals were maintained at constant ambient temperature (23 °C ± 1 °C) under a 12-h light/dark cycle (lights on between 8:00 AM and 8:00 PM) with ad libitum food and water.

### 2.3. ICH Induction and Drug Treatment

An ICH model was prepared as previously described [25]. Briefly, mice were fixed to the stereotaxic instrument under anesthesia by intraperitoneal (i.p.) injection of 0.3 mg/kg medetomidine (FUJIFILM Wako), 4.0 mg/kg midazolam (Sandoz K. K., Tokyo, Japan), and 5.0 mg/kg butorphanol (Vetorphale; Meiji Seika Pharma Co., Ltd., Tokyo, Japan). Then, 0.025 U collagenase type VII (Sigma-Aldrich) dissolved in 0.5 μL saline was injected into the striatum (stereotaxic coordinates; 2.3 mm lateral to the midline, 0.2 mm posterior to the bregma, and 3.5 mm deep below the skull) at a constant rate of 0.2 μL/min. Sham-operated mice were administered the same volume of saline. The body temperature was maintained at 37 °C until waking up from anesthesia. The LXA_4_ receptor ALX/FPR2 agonist, BML-111 (10 mg/mL in methanol), was dissolved in saline at 0.6 mg/mL and administered intravenously at daily doses of 3 mg/kg. The dosage and route of administration of BML-111 were referred to in previous reports; however, they were modified because our preliminary research showed no effects on ICH mice [36]. The administration was performed 20 min before and 24 and 48 h after collagenase injection, for a total of 3 times. The 12/15-lipoxygenase inhibitor, 6,11-dihydro-[1]benzothiopyrano[4,3-b]indole (PD146176) (10 mg/mL in dimethyl sulfoxide), was dissolved in 1% polyoxyethylene sorbitan monolaurate (Tween 20) containing saline at 1 mg/mL and administered intraperitoneally at daily doses of 10 mg/kg.

### 2.4. RNA Isolation and Real-Time Reverse Transcription-Polymerase Chain Reaction (RT-PCR)

Cellular RNA from brain tissue of ICH model mice 24 or 72 h after collagenase injection or treated BV-2 cells was extracted using cold RNAiso Plus (Takara Bio. Inc., Shiga, Japan) and reverse transcribed using a PrimeScript RT Master Mix (Takara). Real-time quantitative PCR was performed using 2 μL complementary DNA and PowerUp^TM^ SYBR^TM^ Green Master Mix (Thermo) in a StepOnePlus™ Real-Time PCR system (Thermo). The thermal cycling program consisted of 95 °C for 120 s to activate the polymerase and 40 cycles at 95 °C for 3 s and 60 °C for 30 s. Reactions were quantified by the comparative threshold cycle method using glyceraldehyde 3-phosphate dehydrogenase (GAPDH) mRNA levels as an internal control. Primer sequences are listed in Table 1.

### 2.5. Immunofluorescence Cell Staining

BV-2 cells were seeded on 35 mm glass bottom dishes (AGC Techno Glass Co., Ltd., Shizuoka, Japan) at 2.0 × 10^4^ cells/cm^2^. The medium was removed 1 h after thrombin treatment and cells were fixed with 4% PFA at room temperature for 30 min. Blocking was performed by soaking in Triton-X 100/PBS (PBST)-containing serum at room temperature for 30 min and then incubation overnight in PBST-containing serum and rabbit anti-NF-κB p65 (1:500, #8242, Cell Signaling Technology, Danvers, MA, USA) as the primary antibody at 4 °C. Cells were incubated in PBST-containing serum, with Alexa Fluor 488-conjugated donkey anti-rabbit IgG (1:500) as a secondary antibody and Hoechst 33,342 as nuclear staining at room temperature for 1 h. Fluorescent images were obtained using an FV-10i (Olympus Corp., Tokyo, Japan) confocal microscope.

### 2.6. Chemotaxis Assay

The chemotaxis assay was performed as previously described [25]. Briefly, dHL-60 cells were resuspended at 5 × 10^5^ cells per 100 μL serum-free RPMI 1640 medium in the upper chambers, while the lower chambers were filled with 480 μL serum-free RPMI 1640 medium plus 120 μL conditioned medium (CM) from thrombin-treated or control BV-2 microglia. LXA_4_ was added to investigate the effect of ALX/FPR2 stimulation; FBS was added to another well as a positive control. After 2 h incubation at 37 °C, cells migrated to the lower chamber were collected and counted using a hemocytometer.

### 2.7. Behavioral Tests

The motor dysfunctions of mice were evaluated by the beam-walking test and modified limb-placing test (MLPT) every 24 h after induction of ICH. These tests were conducted by an experimenter blinded to the treatments. In the beam walking test, mice were trained once daily for 3 days before surgery. Mice were placed on a beam (1.1 m in length, and 1.5 cm in width); the percentage of left hindlimbs stepped off while crossing the beam was obtained as an averaged value from three trials on each day. The MLPT consisted of two limb-placing tasks that assessed the sensorimotor integration of the forelimb and hindlimb by testing their responses to tactile and proprioceptive stimuli. Details for the scoring have been previously described [37].

### 2.8. Immunohistochemical Analysis

Mice were deeply anesthetized with pentobarbital (100 mg/kg, i.p.) 72 h after ICH induction and then transcardially perfused with 30 mL cold phosphate-buffered saline (PBS) and 30 mL of 4% paraformaldehyde (PFA). Obtained brain tissues were postfixed in PFA overnight at 4 °C and soaked in 15% sucrose overnight at 4 °C. The dehydrated brain tissue was frozen, and fixed brains were cut into 30 μm thick coronal sections and mounted onto slides. Antigen retrieval was executed by soaking the sections in 10 mM citrate buffer (pH 8.0) for 30 min at 80–85 °C, followed by cooling at room temperature for 1 h. Blocking was performed by dropping Triton-X 100/PBS (PBST)-containing serum into the sections and allowing it to stand at room temperature for 1 h. Sections were then incubated with PBST-containing serum and primary antibodies overnight at 4 °C. We used the following primary antibodies: rabbit anti-Iba1 antibody (1:1000, 019-19741, FUJIFILM Wako) as a marker of microglia, rabbit anti-human myeloperoxidase (MPO) antibody (1:500, A3098, Dako, Glostrup, Denmark) as a marker of neutrophils, and rabbit anti-FPRL1/RFP antibody (1:200, ab203129, Abcam plc, Cambridge, UK). Anti-MPO antibodies were pre-labeled with biotin according to material data sheet in double staining experiments with anti-FPRL1/RFP antibody (Biotin Labeling Kit, LK03, Dojindo, Kumamoto, Japan). Sections were incubated in PBST-containing serum and Alexa Fluor 488-conjugated donkey anti-rabbit IgG (H+L) (1:500, A21206, Thermo Fisher Scientific Inc., Waltham, MA, USA), Alexa Fluor 555-conjugated donkey anti-rabbit IgG (H+L) (1:500, A31572, Thermo), or Alexa Fluor 555-conjugate streptavidin (1:1000, S21381, Thermo) as secondary antibodies at room temperature for 2 h. Fluorescent images were obtained using a confocal microscope (FV-10i, Olympus). The number of MPO-positive cells was counted using fluorescence images (320 × 320 μm^2^) in the central region of the hematoma. The Iba1-immunopositive area was measured in fluorescence images (640 × 640 μm^2^) containing the hematomal edge using ImageJ software. Double-staining images of ALX/FPR2 and MPO were obtained by NanoZoomer S60 (Hamamatsu Photonics K.K., Hamamatsu, Japan).

### 2.9. Quantification of the Injured Area by Nissl Staining

The 30 µm coronal frozen brain sections obtained every 240 μm were soaked in a 0.1% Cresyl Violet (MP Biomedicals Inc., Santa Ana, CA, USA) solution at 37 °C for 10 min, and then washed and mounted. Images were obtained using a stereomicroscope (SZ61, Olympus Corp.). The injured volume was measured by integrating the Nissl staining negative area using ImageJ software.

### 2.10. Statistical Analysis

All data are presented as mean ± standard error of the mean (S.E.M). Statistical analyses were carried out with GraphPad Prism 7 software (Graph Pad, San Diego, CA, USA). Two group means from normally distributed datasets were compared by the Student’s t-test for independent samples, while two group means from non-normally distributed datasets were compared by the Mann–Whitney U test. Multiple group means from normally distributed datasets were compared by one-way analysis of variance (ANOVA), followed by Tukey’s multiple comparisons tests. The nonparametric Kruskal–Wallis test was used, followed by Dunn’s multiple comparisons test, when the distribution of data points was not suitable for one-way ANOVA. Motor dysfunction data were analyzed by two-way ANOVA with repeated measures followed by Tukey’s multiple comparisons test.

## 3. Results

### 3.1. ALX/FPR2 Stimulation Suppressed the Inflammatory Reactions in Microglia with Thrombin Stimulation

**Figure 1:**
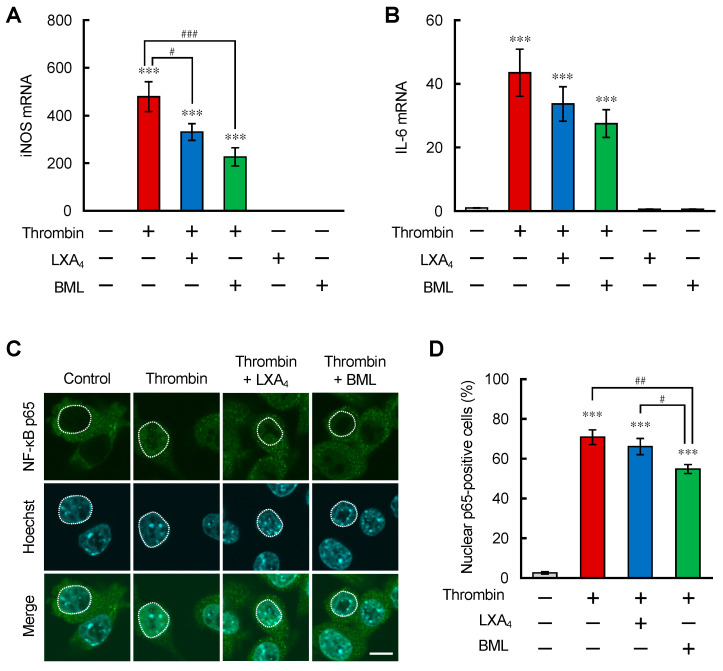
ALX/FPR2 stimulation suppressed inflammatory reactions in BV-2 microglial cells. (**A**,**B**) mRNA expression levels of iNOS (**A**) and IL-6 (**B**) after thrombin treatment (30 U/mL) for 24 h in BV-2 cells measured by qRT-PCR. LXA_4_ (100 nM) and BML-111 (500 nM) were provided 1 h before thrombin treatment. Data shown as fold changes compared with the non-treated group. Mean ± S.E.M., *n* = 9, *** *p* < 0.001 compared with the non-treated group. ^#^ *p* < 0.05, ^###^ *p* < 0.001 compared with the thrombin-treated group. Data were analyzed by one-way ANOVA, followed by Tukey’s multiple comparisons test. (**C**) Representative images of the immunostaining of the NF-κB p65 subunit (green) with nuclear staining (Hoechst 33342; Blue). Cells were treated with thrombin for 1 h and fixed. The white dashed line shows the nucleus. Scale bar = 10 μm (**D**) Quantitative results of the percentage of NF-κB p65-positive cells in the nucleus referred to the total number of cells. Mean ± S.E.M., *n* = 9, *** *p* < 0.001 compared with the control group. ^#^ *p* < 0.05 compared with the LXA_4_ plus thrombin-treated group. ^##^ *p* < 0.01 compared with the thrombin-treated group. Data were analyzed by one-way ANOVA followed by Tukey’s multiple comparisons test.

Thrombin is known as a blood coagulation factor and is increased in the ICH brain. Past reports showed that thrombin stimulated protease-activated receptor-1 (PAR-1) and activated microglial inflammatory reaction in the ICH mouse brain and in murine BV-2 microglial cells. First, we checked the microglial responses to thrombin stimulation in BV-2 cells. Thrombin (30 U/mL) significantly increased the mRNA expression levels of inducible nitric oxide synthase (iNOS) and interleukin-6 (IL-6), peaking at 24 h after treatment (data not shown). Some research reported that stimulation of the lipoxin A_4_ receptor/formyl peptide receptor 2 (ALX/FPR2) expressed on microglia and macrophages attenuated the inflammatory reactions in some conditions. We investigated the effect of ALX/FPR2 stimulation on thrombin-stimulated BV-2 cells. Treatment with LXA_4_ (100 nM) and an ALX/FPR2 agonist, BML-111 (500 nM), 1 h before thrombin treatment, significantly reduced iNOS mRNA expression levels and tended to decrease IL-6 mRNA expression levels (Figure 1A,B). The concentrations of LXA_4_ and BML-111, in some published reports, were adopted as the applicable highest concentration [36,38]. Since the expression levels of proinflammatory molecules, such as iNOS and IL-6, are generally regulated by nuclear factor-kappa B (NF-κB), a transcriptional factor mainly constituted of p65/RelA and p50 subunits, we examined the translocation of the NF-κB p65 subunit into the nucleus in BV-2 cells. NF-κB p65 subunits were expressed in the cytosol in control cells; however, translocated p65 signals were observed in the nucleus of thrombin-treated cells 1 h after treatment. LXA_4_ and BML-111 were provided 1 h before thrombin treatment. LXA_4_ showed no significant decrease compared with the thrombin-treated group; however, BML-111 showed a significant decrease in the percentage of cells showing nuclear p65 compared to the thrombin-treated group and the LXA_4_ plus thrombin-treated group (Figure 1C,D). These results suggest that NF-κB signaling is partially involved in the anti-inflammatory effect of LXA_4_ and BML-111.

### 3.2. LXA_4_ Suppressed the Chemotactic Activity of Neutrophil-Like Cells toward Chemoattractant from Microglia with Thrombin Stimulation

**Figure 2:**
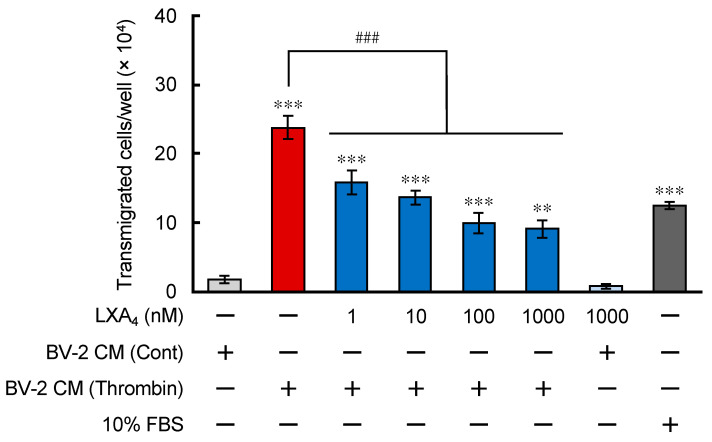
LXA_4_ attenuated the migration of dHL-60 cells toward chemoattractants secreted from BV-2 microglial cells. The chemotactic activity of dHL-60 cells was measured in transwell plates with 3 μm pores. Cells were seeded in the upper chamber and the lower chamber filled with conditioned medium (CM) from untreated or thrombin-treated BV-2 cells. BV-2 cells were treated with thrombin (30 U/mL) and the CM was added to the bottom chamber. LXA_4_ (1–1000 nM) was cotreated with CM from BV-2 cells in bottom chambers. ** *p* < 0.01, *** *p* < 0.001 compared to CM from vehicle-treated BV-2 cells. ^###^ *p* < 0.001 compared with CM from thrombin-treated BV-2 cells. Data were analyzed by one-way ANOVA with Tukey’s post hoc multiple comparisons tests.

After ICH onset, numerous inflammation-inducible cells, such as neutrophils and monocytes, infiltrate into the hematoma by blood leakage. We focused on neutrophils because our findings showed that infiltration of neutrophils defined ICH prognosis. Our previous research revealed that thrombin-treated BV-2 cells secreted some chemoattractant that promoted the migration of neutrophil-like cells. In this case, we applied LXA_4_ under the same experimental conditions. Human promyelocytic leukemia HL-60 cells were maintained in 1% all-trans retinoic acid (ATRA)-containing medium for 5 days and differentiated into neutrophil-like cells (dHL-60 cells) [25]. These cells were seeded on the upper chamber of cell culture inserts with 3 μm pores and the conditioned media (CM) from BV-2 cells treated with thrombin for 12 h was applied to the bottom chamber as previously reported. Transmigrated cells were counted in the bottom chamber for 2 h after seeding. CM from thrombin-treated BV-2 cells promoted the migration of dHL-60 cells compared with CM from control BV-2 cells. LXA_4_ (1–1000 nM) was cotreated in the bottom chamber with CM from thrombin-treated BV-2 cells. LXA_4_ significantly decreased the number of transmigrated dHL-60 cells in the bottom chamber at all concentrations (Figure 2). We also reported that dHL-60 cells expressed ALX/FPR2 [25]. These data suggest that the stimulation of ALX/FPR2 has suppressive effects on neutrophil migration in ICH conditions.

### 3.3. ALX/FPR2 Was Expressed on Neutrophils and Its Expression Was Increased in the ICH Mouse Brain

**Figure 3:**
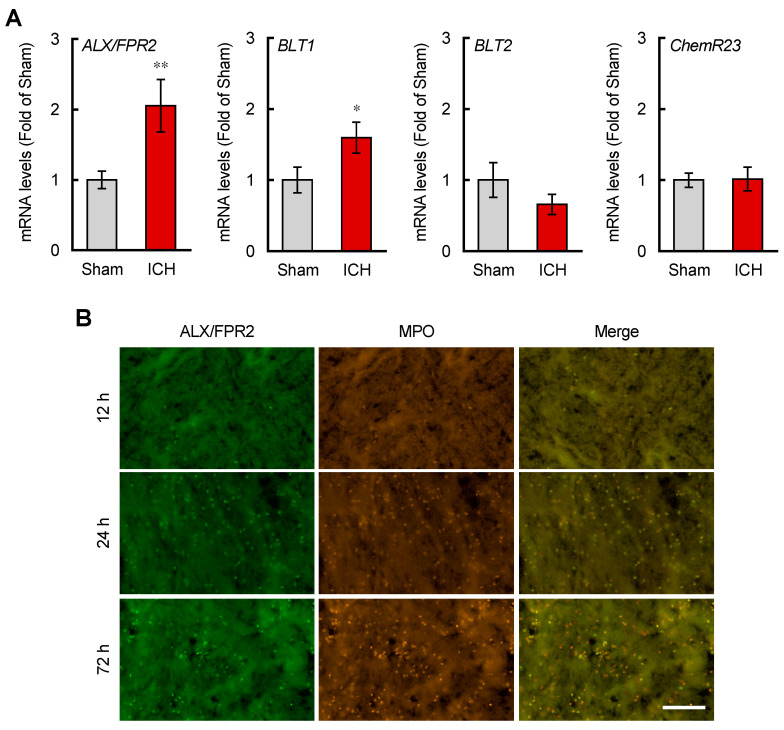
Upregulation of ALX/FPR2 in the brain of ICH model mice. (**A**) Quantitative analysis of ALX/FPR2, BLT1, BLT2, and ChemR23 mRNA expression levels in the ipsilateral 4 mm thickness brain tissues containing hematoma at 24 h after ICH induction. Data were normalized with GAPDH mRNA levels as the internal control and shown as fold changes compared with the sham-operated group. Mean ± S.E.M., *n* = 13, * *p* < 0.05, ** *p* < 0.01 compared with sham group. Data were analyzed by the nonparametric Mann–Whitney U test. (**B**) Representative images co-labeled with ALX/FPR2 (Green) and MPO (Orange) in the center region of the hematoma at indicated times after ICH induction. Scale bar = 100 µm.

ALX/FPR2 is widely expressed in many central nervous system (CNS) cell types and peripheral tissues; however, RNA sequencing data have shown a low expression level of ALX/FPR2 in the CNS [39]. We analyzed the expression levels of ALX/FPR2 and related receptors in ICH conditions. ALX/FPR2 and BLT1, but not BLT2 and ChemR23 mRNA levels were significantly increased 24 h after ICH induction (Figure 3A). Immunohistochemical analysis revealed that ALX/FPR2 was strongly expressed in hematoma and colocalized with myeloperoxidase (MPO)-positive cells (Figure 3B). The number of ALX/FPR2-positive cells increased with the same time-course of MPO-positive cells; the ALX/FPR2 mRNA increase may reflect the number of MPO-positive neutrophils. Considering the expression pattern of ALX/FPR2, ALX/FPR2-expressing neutrophils were considered the therapeutic target for ICH.

### 3.4. BML-111 Attenuated the Motor Coordination Dysfunctions in ICH Mice

**Figure 4:**
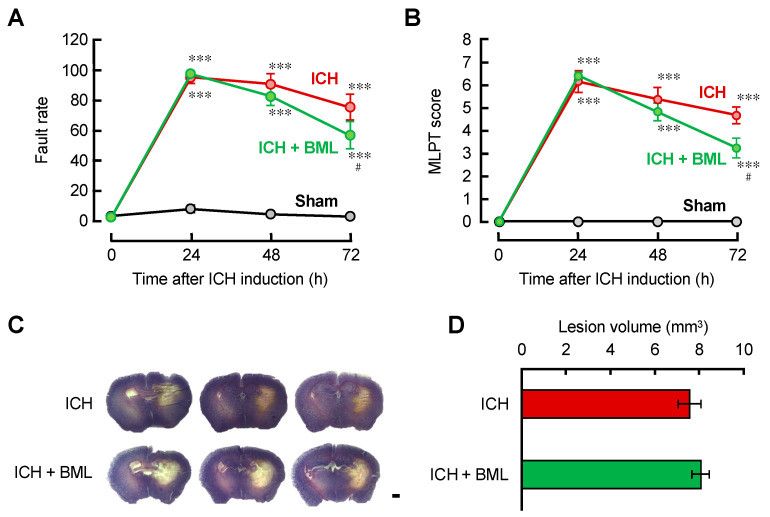
The ALX/FPR2 agonist BML-111 improved motor dysfunction associated with ICH. (**A**,**B**) Motor functions were evaluated by the beam-walking test (**A**) and modified limb-placing test (**B**) every 24 h after ICH induction. Mean ± S.E.M., *n* = 14–15. *** *p* < 0.001 compared with the sham-operated group at the same time points. ^#^ *p* < 0.05 compared with the ICH group. Data were analyzed statistically by two-way ANOVA with repeated measures followed by Tukey’s multiple comparisons test. (**C**) Three representative sections of Nissl staining at 72 h after ICH induction. Scale bar = 2 mm. (**D**) Quantitative results of the lesion volume calculated from the Nissl-negative region in every 240 μm series tissue section. Mean ± S.E.M., *n* = 7–9.

We investigated the effect of ALX/FPR2 signaling in ICH model mice. Because ALX/FPR2 stimulation inhibited the neuroinflammatory reaction in BV-2 cells and neutrophil chemotaxis, we focused on the effect of intrinsic LXA_4_ produced in the brain. LXA_4_ is produced from arachidonic acid; several enzymes are involved in its production, including 12/15-lipoxygenase (12/15-LOX) as a rate-limiting enzyme. In some cases, deletion or inhibition of 12/15-LOX affected pathophysiological events; however, there were no reports about its effects on ICH. PD146176 (10 mg/kg), an inhibitor of 12/15-LOX, was intraperitoneally injected into ICH mice daily; however, there were no beneficial effects (data not shown). We previously provided the quantification of arachidonic acid metabolites, including LXA_4_ and LTB_4_; however, LXA_4_ was under the detection limit in the ICH brain. Since the contribution of endogenous LXA_4_ to ICH pathophysiology was low, BML-111, an ALX/FPR2 agonist, was administered to ICH model mice. In this study, LXA_4_ was not used in in vivo experiments because it is rapidly metabolized in the body. BML-111 was intravenously administered at a dose of 3 mg/kg because there were no beneficial effects by its intraperitoneal administration at 1 mg/kg, which was reported as the highest dose in previous experiments [36]. The beam-walking test measured the percentage of stepping off the disabled side limb from the narrow stainless pole. MLPT measured the disability score composed of three evaluation criteria, as we reported [37]. Motor coordination dysfunction appeared, peaking at 24 h after ICH induction, and gradually recovered in each test. Daily BML-111 (3 mg/kg) administration significantly attenuated motor dysfunction at 72 h after ICH induction (Figure 4A,B). Hematoma expansion/resolution is one of the major pathological events to reflect ICH outcomes, not only in animal models, but also in patients with ICH. In animal experiments, Nissl staining was used, which visualizes the intact brain tissues. The Nissl-negative region showing the lesion area and volume almost matched with the hematoma volume. We tested the effect of BML-111 on lesion volume 72 h after ICH induction; however, BML-111 did not affect the lesion volume (Figure 4C,D).

### 3.5. BML-111 Attenuated Neuroinflammation in the ICH Mouse Brain

**Figure 5:**
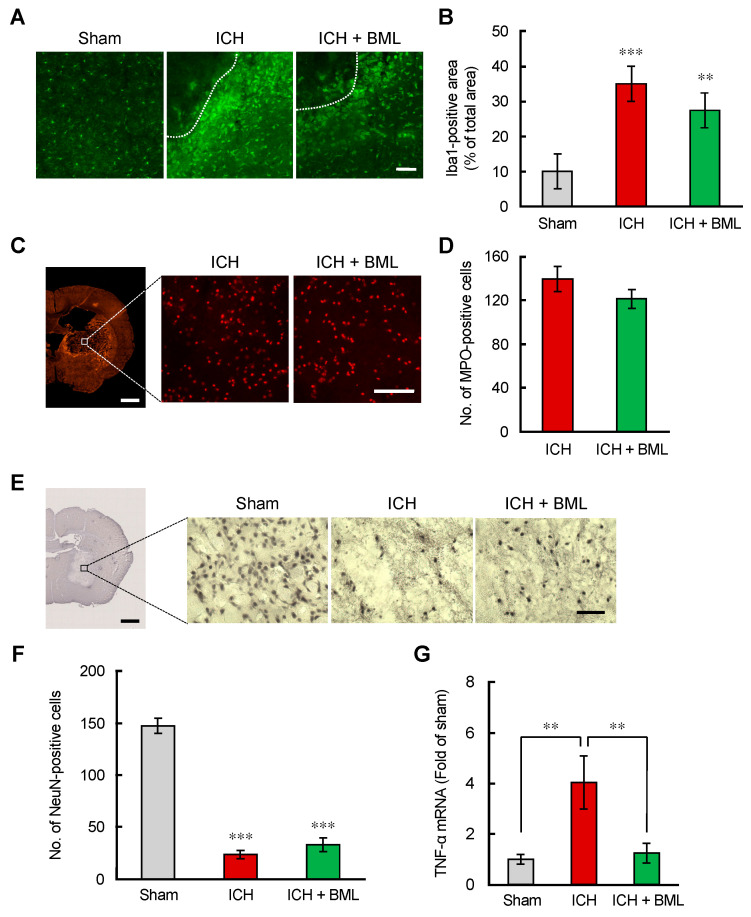
The ALX/FPR2 agonist BML-111 exerted mild attenuation of neuroinflammation 72 h after ICH induction. (**A**,**C**,**E**) Representative images of Iba-1 (**A**), MPO (**C**), and NeuN (**E**)-immunopositive cells in the peripheral region of hematoma 72 h after ICH induction. Dashed lines are the edge of the hematoma. Scale bar = 100 μm (**A**,**C**), 50 µm (**E**), 1 mm in low-magnification images (**C**,**E**). (**B**,**D**,**F**) Quantification of the Iba-1-immunopositive region in the images (640 × 640 μm^2^) (**B**), the number of MPO-immunopositive cells in the images (320 × 320 μm^2^) (**D**), and the number of NeuN-immunopositive cells in the images (230 × 190 μm^2^) (**F**). ** *p* < 0.01, *** *p* < 0.001 compared to those in the sham-operated group. Mean ± S.E.M., *n* = 7–9 (**B**), 7 (**D**), and 8–9 (**F**). Data were analyzed by one-way ANOVA with Tukey’s post hoc multiple comparisons tests. (**G**) Quantitative analysis of mRNA expression levels of TNF-α in the ipsilateral 4 mm thickness brain tissues containing hematoma at 72 h after ICH induction. Mean ± S.E.M., *n* = 4–6. ** *p* < 0.01 compared with the ICH group. Data were statistically analyzed by one-way ANOVA followed by Tukey’s multiple comparisons test.

Neuroinflammation is a characteristic pathological change after ICH onset. Microglia/macrophages are activated and proliferate around the hematoma, and neutrophils and lymphocytes invade the brain through bleeding. ALX/FPR2 stimulation affects microglia and neutrophils under in vitro conditions (Figure 1 and Figure 2). We evaluated the effects of BML-111 against microglia and neutrophils in ICH mice brains. Brain tissues were collected 72 h after ICH induction, a microglial activation peak time. To investigate its effect on microglial activation, immunostaining for Iba1, the microglial marker, was performed. The activation of microglia was confirmed by morphological changes from bushy to round shapes around the hematoma (Figure 5A). Quantification data showed that the Iba1-immunopositive area was increased by ICH induction. BML-111 slightly, but not significantly, reduced the area (Figure 5B). MPO-positive cells were observed in the central region of hematoma at 24 and 72 h (Figure 3). BML-111 did not reduce the number of MPO-positive cells 72 h after ICH induction (Figure 5C,D). Neuroinflammation causes neuronal damage and neuronal loss. Neuronal nuclei (NeuN)-positive cells were severely reduced in the central region of the hematoma; however, BML-111 did not show any changes (Figure 5E,F). Finally, we investigated the mRNA expression levels of TNF-α, because activated microglia/macrophages and neutrophils excessively produce proinflammatory cytokines. ICH induction prominently increased the expression levels of TNF-α mRNA at 72 h after ICH induction. BML-111 completely suppressed the increase in TNF-α mRNA (Figure 5G). These data suggest that systemic injection of BML-111 could not reduce the number of activated microglia and neutrophils, but could suppress their inflammatory phenotypes.

## 4. Discussion

Neuroinflammation is a common key pathological change in neurodegenerative disorders and stroke [40]. Increasing lines of evidence proposed that reducing the excessive glial activation and the infiltration of peripheral tissue-derived inflammatory cells, such as neutrophils, macrophages, and lymphocytes, is an effective therapeutic strategy of ICH [41]. In particular, many molecules expressed on microglia were reported as therapeutic targets, such as toll-like receptor 4 (TLR4), receptor for advanced glycation endproducts (RAGE), and C-C motif chemokine receptor 2 (CCR2) [11,19]. These receptors are also expressed on neutrophils, but previous research mainly focused on microglial responses. Recent studies revealed that neutrophil deprivation by the injection of anti-polymorphonuclear (PMN) antibodies attenuated axonal damages in the ICH mouse brain [14]. Therefore, we assumed that both microglia and neutrophils should be regulated under ICH conditions. Our recent studies focused on the molecules that can regulate microglia/macrophages and neutrophils. The LTB_4_ receptor blockade reportedly inhibited microglial inflammatory reactions and suppressed neutrophil migration activities [23]. Our previous research revealed that LTB_4_ was involved in the progression of ICH pathological changes and that LTB_4_ was released from thrombin-stimulated microglia [25]. LXA_4_ was produced from AA by a similar pathway with LTB_4_; however, LXA_4_ was reported to have anti-inflammatory effects on microglia/macrophages and a suppressive effect on neutrophil migration, in contrast to LTB_4_ [32]. Accordingly, we investigated the efficacy of LXA_4_-ALX/FPR2 signaling as a therapeutic target for ICH.

BML-111, an ALX/FPR2 agonist, suppressed IL-6; iNOS mRNA expression induced by thrombin stimulation in microglia (Figure 1). BML-111 could partially suppress the nuclear translocation of NF-κB, consistent with past reports that an LXA_4_ analog exerted an anti-inflammatory effect by inhibiting the NF-κB pathway [42]. Stimulation of ALX/FPR2 signaling also suppressed the transmigration of neutrophil-like dHL-60 cells (Figure 2). The suppressive effect was stronger than the effect of the LTB_4_ receptor antagonist that we previously showed [25]. In ICH conditions, many molecules that promote neutrophil migration were reported; however, suppressive molecules were not [43,44]. CM from thrombin-stimulated BV-2 may contain many chemoattractant factors, such as LTB_4_, CCL2, and CXCL2. Therefore, the effect of antagonism of the LTB_4_ receptor was restricted; however, the effect of stimulation of ALX-FPR2 may be strong. These data suggest that LXA_4_-ALX/FPR2 signaling has beneficial effects both on microglia/macrophages and neutrophils in the ICH brain.

The receptor for LXA_4_ was well researched in peripheral tissues, but not in the CNS [45]. We analyzed the expression profiles of ALX/FPR2 in the ICH brain. The level of ALX/FPR2 mRNA significantly increased 24 h after ICH. This change might reflect the number of neutrophils infiltrated by bleeding, because co-staining data with ALX/FPR2 only merged with MPO in the central region of the hematoma. These data suggest that microglial ALX/FPR2 expression was considerably small and that neutrophils mainly express ALX/FPR2. Expression changes of BLT1 and BLT2 mRNA were consistent with our previous research that BLT1 predominantly increased compared with BLT2 in the ICH brain [23]. These changes also reflected the number of neutrophils because BLT1 is known to express on neutrophils. ChemR23, a receptor for resolvin E1 that is expressed in microglia/macrophages, was not changed. These expression profiles of ALX/FPR2, BLT1, BLT2, and ChemR23 suggest the efficacy of focusing neutrophils as the therapeutic target in the ICH brain. Some previous reports showed that stimulation of ALX/FPR2 by lipoxin A_4_ methyl ester or annexin A1 attenuated neuroinflammation and motor dysfunction in ICH mice; however, there are no data on the migration of peripheral tissue-derived cells such as neutrophils [46,47].

The therapeutic effects of BML-111 in the ICH mouse model were low and showed mild suppression of motor dysfunction 72 h after ICH induction without changes in lesion volume (Figure 4). In the ICH brain, neuroinflammatory reactions peaked at 72 h after ICH induction using the number of activated microglia and the number of neutrophils as inflammatory indexes. BML-111 might suppress neuroinflammation at 72 h but not affect other pathogenic events at an early point. Unfortunately, the effects of BML-111 on the number of Iba-1-positive microglia and the number of MPO-positive neutrophils were not significant (Figure 5). There are two possible reasons for the small effect of BML-111. First, it might be caused by the low expression of ALX/FPR2 in microglia. Second, it might be due to the lack of differences in the concentration gradient of BML-111 between the CNS and peripheral tissues, because BML was injected systemically. In contrast, BML-111 significantly suppressed the mRNA expression of TNF-α 72 h after ICH induction (Figure 5). Neutrophils are known to produce ROS, nitrogen oxide, and cytokines such as TNF-α. BML-111 might affect the inflammatory functions of neutrophils without changes in the number of cells in the hematoma. To address the small efficacy of BML-111 on the pathophysiological changes in the ICH brain, we have to optimize the therapeutic regimens. In this report, we showed ALX/FPR2 localized on neutrophils but not microglia/macrophages. Upregulation of ALX/FPR2 on microglia/macrophages may exert potentiation of anti-inflammatory effects of BML-111 because our in vitro experiments showed that stimulation of ALX/FPR2 demonstrated anti-inflammatory effects. Furthermore, an efficient drug delivery system may improve the effects of BML-111. Recently, drug administration via the nasal cavity has been well researched because intranasal administration offers advantages over a systemic drug delivery system, as it directly delivers the drug into the brain via the olfactory route [48]. We should find the strategy potentiating the effect of the agonist of ALX/FPR2 for the ICH therapy in our future research.

Overall, LXA_4_-ALX/FPR2 signaling can suppress the inflammatory phenotypes of microglia/macrophages and migration activities of neutrophils. In this context, we showed small therapeutic effects in the ICH model mice, because of pharmacokinetic insufficiency. The specific upregulation of LXA_4_ in the hematoma may exert potent anti-neuroinflammatory responses via the suppression of microglia/macrophage activation and migration of neutrophils. Furthermore, we previously reported that the suppression of LTB_4_ signaling alleviated the neuroinflammation after ICH [23]. These findings suggest that AA metabolism regulation, such as the combination of LTB_4_ signaling downregulation and LXA_4_ signaling upregulation, may have potent therapeutic effects for ICH.

## 5. Conclusions

In summary, we found that the LXA_4_-ALX/FPR2 signal alleviated the neuroinflammation associated with ICH and slightly improved motor function in a mouse model of ICH. Considering that LTB4 is involved in the exacerbation of ICH [23], it is suggested that the regulation of lipid metabolism after ICH strongly decides the pathological conditions. Further research focused on the regulation of lipid metabolism will lead to the development of novel therapeutic agents for ICH.

## Figures and Tables

**Table 1:** Primer sequences for quantitative RT-PCR.

Gene	Primer Sequences	
TNF-α	Fw	5′-TTCTGTCTACTGAACTTCGGGGTGATCGGTCC-3′
	Rv	5′-GTATGAGATAGCAAATCGGCTGACGGTGTGGG-3′
IL-6	Fw	5′-TCCAGTTGCCTTCTTGGGAC-3′
	Rv	5′-GTGTAATTAAGCCTCCGACTTG-3′
iNOS	Fw	5′-TGCTTTGTGCGAAGTGTCAGT-3′
	Rv	5′-CGGACCATCTCCTGCATTTCT-3′
ALX/FPR2	Fw	5′-GCTGGTTTCCCTTTCAGCTTGTG-3′
	Rv	5′-AATCCTCACTCAGGGCTCTCTCA-3′
BLT1	Fw	5′-GTCTGGACCGATCACTGGCA-3′
	Rv	5′-TGGGATAGTTCGGAGCGCAG-3′
BLT2	Fw	5′-ACAGCCTTGGCTTTCTTCAG-3′
	Rv	5′-TGCCCCATTACTTTCAGCTT-3′
ChemR23	Fw	5′-AGTGGGGTTCCCAAGTGTGG-3′
	Rv	5′-GCACGGCGACATCGTGTATG-3′
GAPDH	Fw	5′-ACCATCTTCCAGGAGCGAGA-3′
	Rv	5′-CAGTCTTCTGGGTGGCAGTG-3′

## Data Availability

The datasets in this study are available by request to the corresponding author.
